# Supplementary material for: Idiosyncratic Purifying Selection on Metabolic Enzymes in the Long-Term Evolution Experiment with Escherichia coli
Source: Genome Biol Evol. 2022 Aug 17;14(12):evac114. doi: 10.1093/gbe/evac114 (PMC9768419; doi:10.1093/gbe/evac114)
Supplement: evac114_Supplementary_Data [file evac114_supplementary_data.zip › LTEE-STIMS-metabolism-v2-Supplementary-Information.docx]

**SUPPLEMENTARY INFORMATION**

**Supplementary File 1: Mapping of REL606 genes into the four gene sets described in Figure 1.**

**Supplementary Figure 1. The result of running STIMS on BiGG core enzymes and superessential enzymes (nonmutator populations only).** Each panel shows the cumulative number of mutations in the gene set of interest normalized by the combined length of that gene set (solid line), in the six nonmutator LTEE populations. For comparison, random sets of genes (with the same cardinality as the gene set of interest) were sampled 1,000 times, and the cumulative number of mutations in those random gene sets, normalized by gene length, was calculated. The middle 95% of this null distribution is shown as shaded points. When a solid line falls below the shaded region, then the gene set of interest shows a significant signal of either relaxed or purifying selection (*p* < 0.025 for a one-tailed test). For further details, see Figure 2 of Maddamsetti and Grant (2022), which illustrates how STIMS works. A) The result of running STIMS on enzymes in the BiGG *E. coli* core metabolism model. B) The result of running STIMS on enzymes catalyzing superessential metabolic reactions.


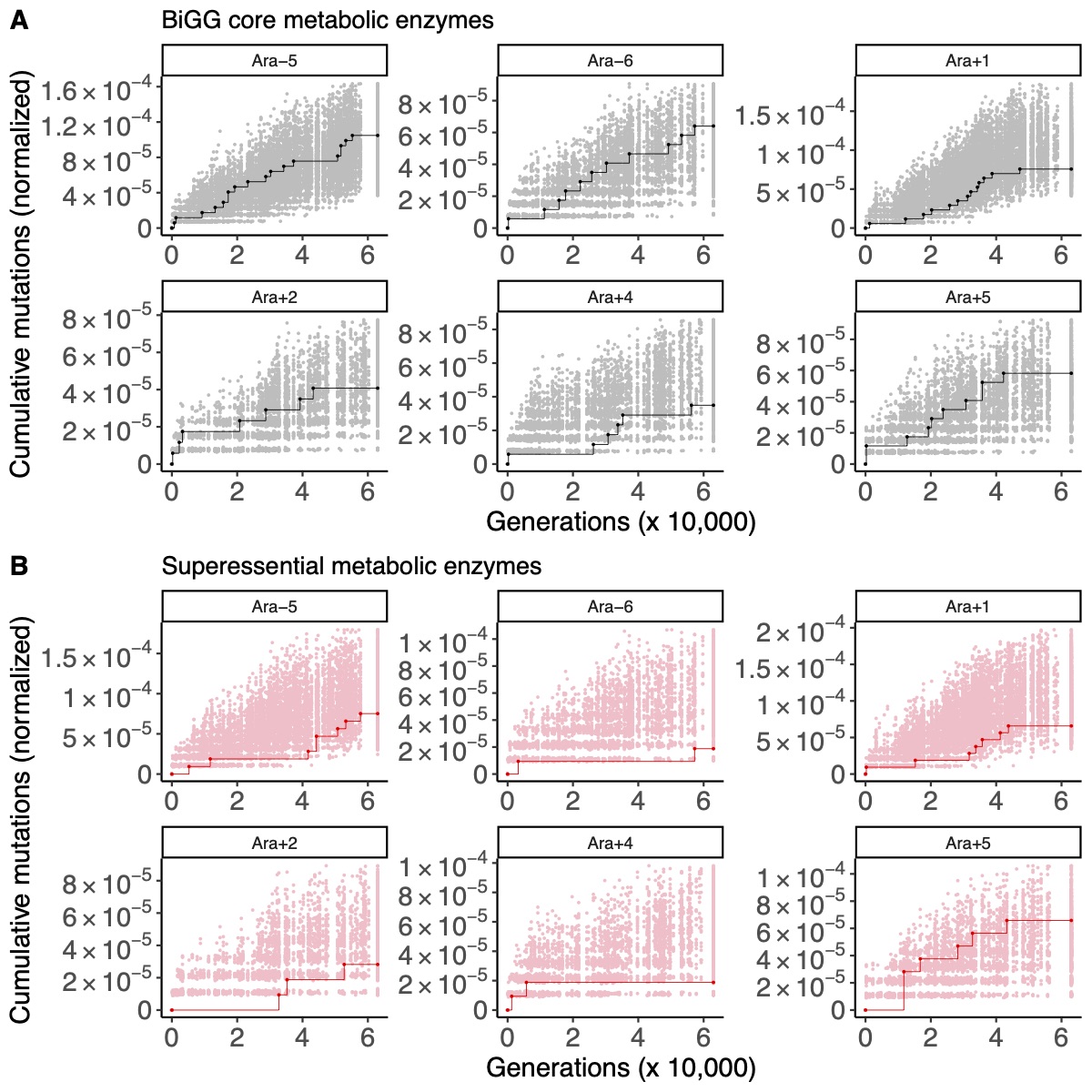


**Supplementary Figure 2. The result of running STIMS on specialist enzymes and generalist enzymes (nonmutator populations only).** Each panel shows the cumulative number of mutations in the gene set of interest normalized by the combined length of that gene set (solid line), in the six nonmutator LTEE populations. For comparison, random sets of genes (with the same cardinality as the gene set of interest) were sampled 1,000 times, and the cumulative number of mutations in those random gene sets, normalized by gene length, was calculated. The middle 95% of this null distribution is shown as shaded points. When a solid line falls below the shaded region, then the gene set of interest shows a significant signal of either relaxed or purifying selection (*p* < 0.025 for a one-tailed test). For further details, see Figure 2 of Maddamsetti and Grant (2022), which illustrates how STIMS works. A) The result of running STIMS on specialist enzymes. B) The result of running STIMS on generalist enzymes.

**
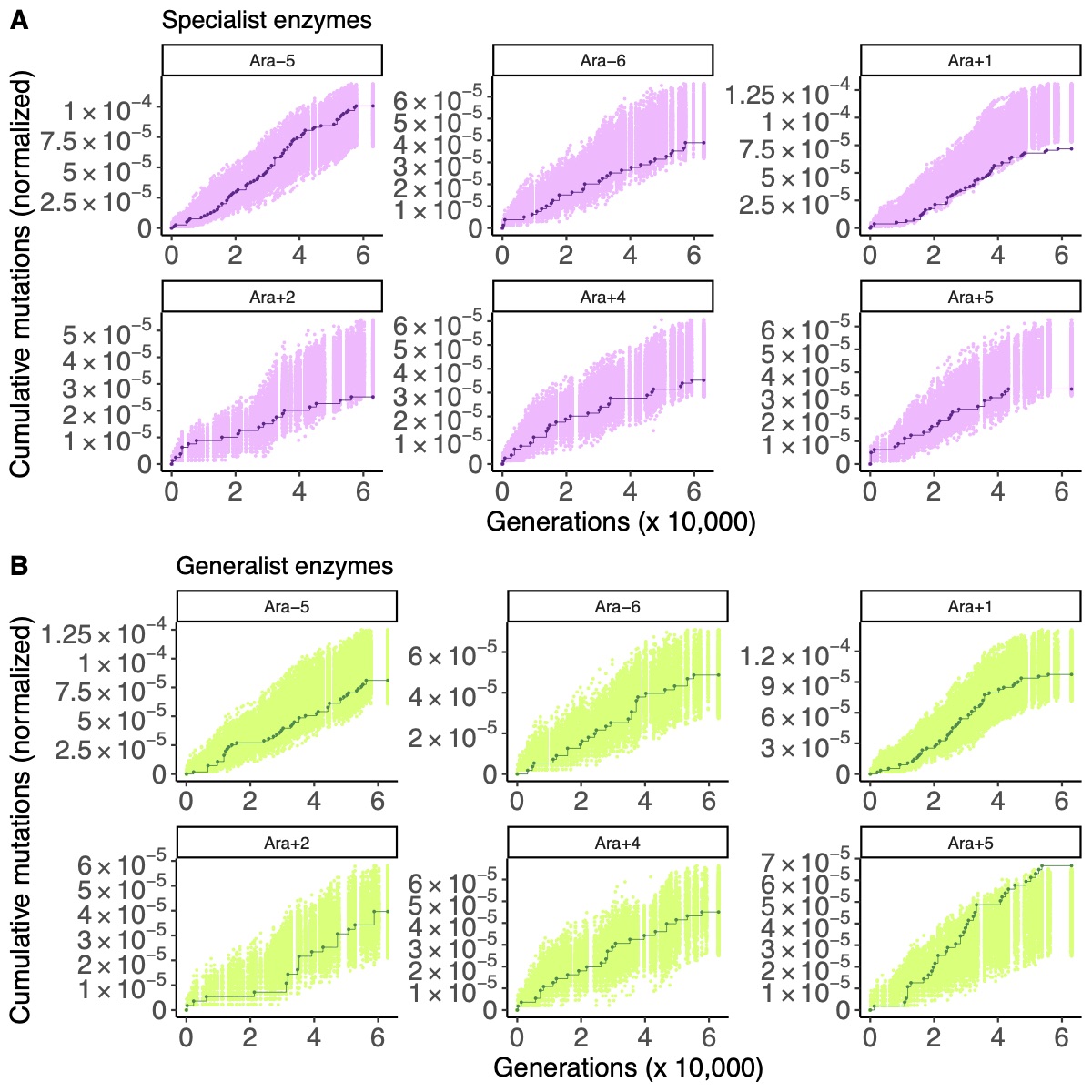
**

**Supplementary Figure 3. The result of running STIMS on core genes in all 1,000 minimal genomes (nonmutator populations only)**. Each panel shows the cumulative number of mutations in the gene set of interest normalized by the combined length of that gene set (solid line), in the six nonmutator LTEE populations. For comparison, random sets of genes (with the same cardinality as the gene set of interest) were sampled 1,000 times, and the cumulative number of mutations in those random gene sets, normalized by gene length, was calculated. The middle 95% of this null distribution is shown as shaded points. When a solid line falls below the shaded region, then the gene set of interest shows a significant signal of either relaxed or purifying selection (*p* < 0.025 for a one-tailed test). For further details, see Figure 2 of Maddamsetti and Grant (2022), which illustrates how STIMS works.

**
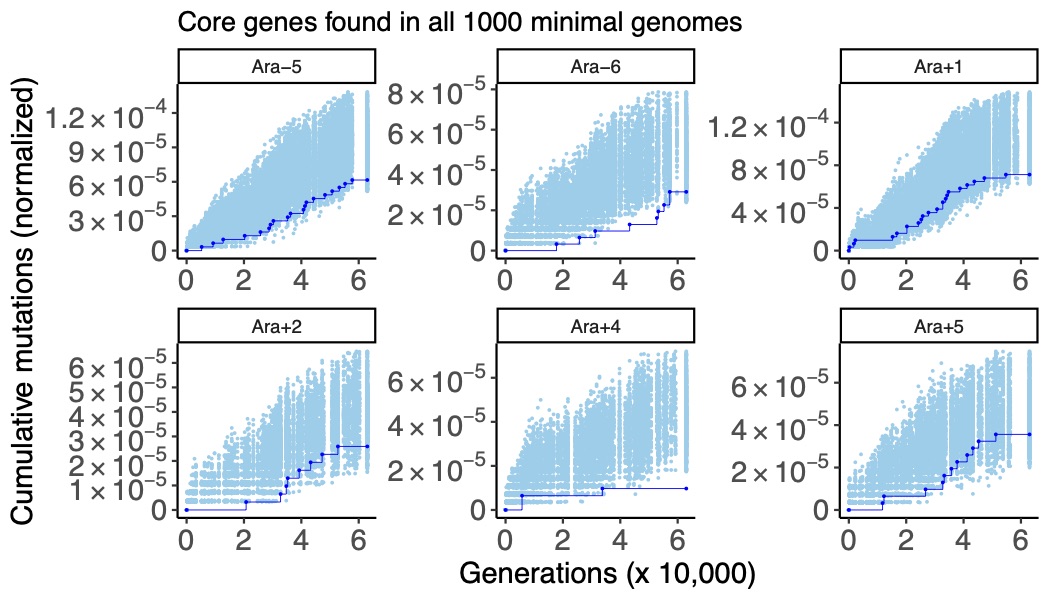
**
